# The β-globin Replicator greatly enhances the potential of S/MAR based episomal vectors for gene transfer into human haematopoietic progenitor cells

**DOI:** 10.1038/srep40673

**Published:** 2017-01-20

**Authors:** Eleana F. Stavrou, Vassileios M. Lazaris, Aristeidis Giannakopoulos, Eirini Papapetrou, Alexandros Spyridonidis, Nikolas C. Zoumbos, Antonis Gkountis, Aglaia Athanassiadou

**Affiliations:** 1 Department of General Biology, School of Medicine, University of Patras, Greece; 2 Haematology Unit Department of Internal Medicine, School of Medicine, University of Patras, Greece; 3 Gene and Cell Therapy Center, Haematology Department-BMT Unit, George Papanicolaou Hospital, Thessaloniki, Greece

## Abstract

Specific human chromosomal elements enhance the performance of episomal gene-transfer vectors. S/MAR-based episomal vector pEPI-eGFP transfects CD34^+^ haematopoietic cells, but only transiently. To address this issue we reinforced (1) transgene transcription by replacing the CMV promoter driving eGFP with the EF1/HTLV or SFFV promoters to produce vectors pEPI-EF1/HTLV and pEPI-SFFV, respectively; and (2) plasmid replication by inserting the replication-Initiation Region (IR) from the β-globin locus into vector pEPI-SFFV to produce vector pEP-IR. All vectors supported stable transfections in K562 cells. Transfections of CD34^+^ cells from peripheral blood of healthy donors reached 30% efficiency. Upon evaluation of CD34^+^/eGFP^+^ cells in colony-forming cell (CFC) assays, vector pEP-IR showed superior performance after 14 days, by fluorescent microscopy: 100% eGFP^+^-colonies against 0% for pEPI-eGFP, 56.9% for pEPI-SFFV and 49.8% for pEPI-EF1/HTLV; 50% more plasmid copies per cell and 3-fold eGFP expression compared to the latter two constructs, by quantitative (q)PCR and RT-qPCR, respectively. Importantly, the establishment rate in CFC assays was 15% for pEP-IR against 5.5% for pEPI-SFFV and 5% for pEPI-EF1/HTLV. Vector pEP-IR shows extremely low delivery rate but supports eGFP expression in thalassaemic mouse haematopoietic progenitor cells. The IR is a novel human control element for improved episomal gene transfer into progenitor cells.

The design and use of extrachromosomal vectors, suitable for efficient and stable transfection of haematopoietic progenitor cells, is an important goal for the gene therapy of haemoglobinopathies. The development of extrachromosomal vectors has mainly been driven by the need to address the safety issue of gene therapy vectors, in particular, the problem of insertional mutagenesis^1^, and involves vectors such as self-replicating stable episomes^2^, pFARs-plasmids free of antibiotic resistance markers^3^, and minicircle DNA plasmid derivatives lacking a bacterial backbone^4^. The presence of the scaffold/matrix attachment region (S/MAR) also confers long-term mitotic stability to integration-deficient lentiviral, episomal vectors^5^,^6^; however, that of the truncated S/MAR does not improve episomal retention^7^.

Key issues in the development of episomal vectors are currently the establishment in the host nucleus^8^,^9^, the transgene expression^10^,^11^ and the delivery in progenitor cells^10^. The prototype episomal vector pEPI-1^2^ does not code for any viral protein, and it contains the S/MAR from the 5′ end of the human β-interferon gene^2^, an element that facilitates the vector’s nuclear retention.

The S/MARs are AT rich chromosomal elements that play a role in chromatin boundary formation^12^ and bind to SAF-A protein^13^, mediating the tethering of pEPI-1 plasmid to the nuclear matrix. A prerequisite for the S/MAR to exert its function is to be transcribed^14^. The S/MAR in pEPI-1 is part of the pCMV-GFP-S/MAR transcription cassette, which contains the GFP reporter gene, driven by the pCMV – the cytomegalovirus immediate-early promoter – so that transcription runs into S/MAR. pEPI-1 is maintained in low copy numbers, 2 to 12 episomes per cell^15^,^16^. It replicates once per cell cycle synchronously with cellular DNA, with the elements of the replication machinery assembling on many, probably random, sites along its DNA^17^ even in the absence of the SV40 origin^18^.

pEPI-eGFP, derived from pEPI-1 by replacement of the GFP by eGFP^17^, functions as an episome (i) *in vitro,* in several cell lines and primary cell cultures^19^, (ii) *in vivo*, in the mouse liver^20^ and (iii) in genetically modified pigs^21^. Other modifications of the pEPI-1 system with improved performance include the minicircle^22^, free of prokaryotic parts and drug selection markers and thus resisting silencing, as well as the vector pEPito^23^, which is depleted of CpG motives. Recently, tumour- and tissue-specific episomal vectors have been developed^24^ and a high capacity hybrid vector – HCAdV-pEPito, based on adenoviral and pEPito vectors and combining high level of vector delivery with episomal status - has been demonstrated to be efficient in *in vitro* as well as in *in vivo* studies^25^. However, as part of another plasmid, namely pCEP4, the S/MAR functions in a context-dependent manner^26^, and imposes restrictions in plasmid DNA replication, resulting in episome loss^27^. In the same study, plasmid DNA replication was restored by the introduction of yet another chromosomal element, namely the β-globin Replicator, a *bona fide* mammalian Replicator from the human β-globin locus^28^,^29^.

The vector pEPI-eGFP has been shown to mediate efficient and stable transfection in the haematopoietic cells K562^19^, as does its β-globin derivative^30^. It is also capable of efficient delivery into human CD34^+^ cells, albeit with inefficient long-term retention not exceeding 1% of cells^19^. Vector pEPI-eGFP, therefore, is not appropriate for gene transfer into human, haematopoietic progenitor cells, and modifications are needed to restore its function in these cells. Such modifications may aim at enhancing transcription running through the S/MAR or/and enforcing the plasmid’s replication potential.

We herein present the development of episomal vectors, derivatives of pEPI-eGFP, capable of mediating efficient and potentially stable transfection in haematopoietic, progenitor cells. This is achieved by the use of the replication-Initiation Region (IR) from the human β-globin locus containing the 1.3 kb that represents the consensus IR region^28^. This replication-Initiation Region (IR) is considered to be a Replicator, in the sense that it is capable of initiating DNA replication in non- native sites^28^,^29^, and it is referred to, hereafter, as “β-globin Replicator” or, simply, as “IR”.

## Results

Our first objective was to ensure transcription through eGFP-S/MAR in CD34^+^ cells. In vector pEPI-eGFP, transcription through eGFP-S/MAR is driven by promoter pCMV, a strong promoter in a variety of cells, but with variable activity in haematopoietic progenitor cells. In our experience, pCMV-driven eGFP expression within the pEPI-eGFP episomal vector is very low in the progeny of cord blood CD34^+^ cells and detectable only by RT-PCR^20^. As transcription of the pCMV-eGFP-S/MAR cassette is a prerequisite for S/MAR function, we hypothesized that vector performance could be improved by the replacement of pCMV by promoters that are active in human haematopoietic progenitor cells, such as the hybrid promoter Elongation Factor 1α/human T-cell leukemia virus (EF1α/HTLV)^31^ (Genbank accession number HG530137.1) and the U3 region of the long terminal repeat (LTR) from spleen focus -forming virus (SFFV)^32^ (Genbank accession number AJ224005.1).

Our second objective was to reinforce the DNA replication capacity of these plasmids, which may contribute to higher rate of episome establishment. As mentioned previously, in our experience, the inclusion of the β-globin Replicator- (see Suppl Fig. S0) can rescue episome’s replication and can result in higher episome retention^27^. We therefore used the β-globin Replicator in a plasmid with altered promoter, in order to investigate its performance as part of S/MAR-pEPI-eGFP based vectors.

### Constructs and Stress-Induced Duplex Destabilization (SIDD) analysis

**Figure 1:**
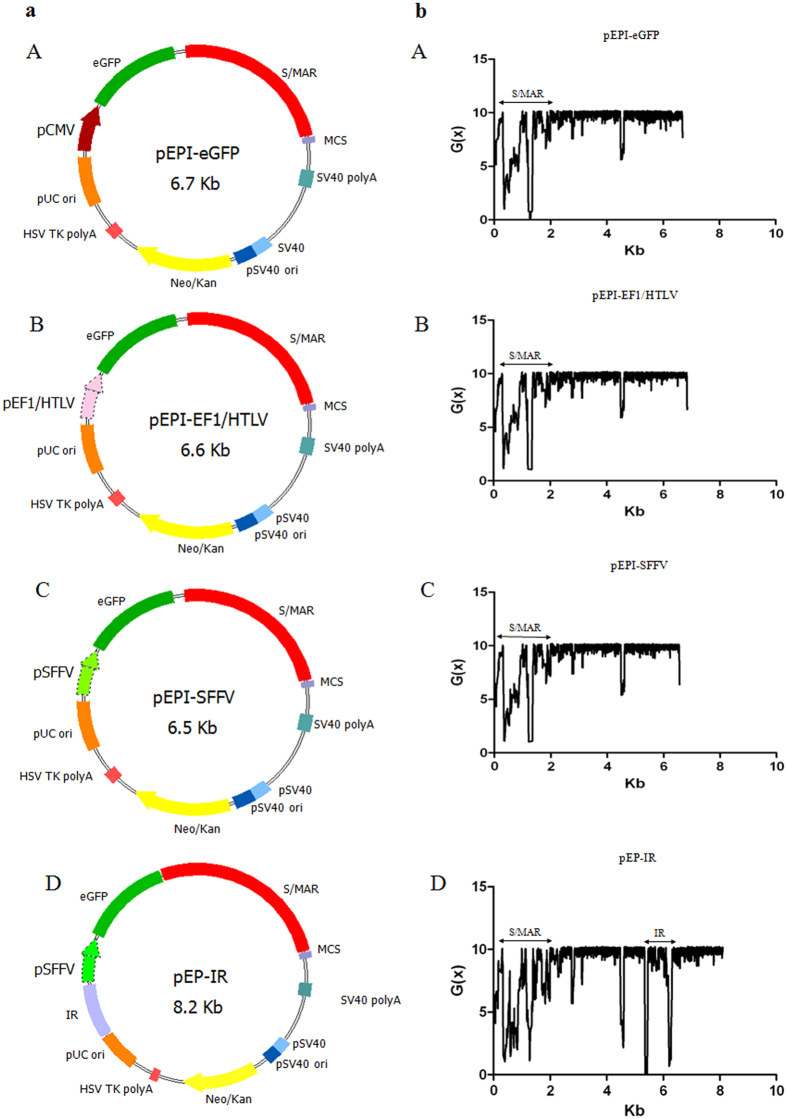
Plasmid constructs and corresponding SIDD profiles. Column (**a**) shows control plasmid pEPI-eGFP, constructs pEPI-EF1/HTLV and pEPI-SFFV containing promoter EF1/HTLV and SFFV respectively, in place of the CMV promoter driving eGFP expression and construct pEP-IR with the insertion of the IR 5′ to the SFFV promoter. Column (**b**) shows the corresponding SIDD profiles of the plasmids used. G(x): destabilization energy (for explanation see Methods). All SIDD profiles show the highly destabilized area of S/MAR; the lightly destabilized site at 4.7 kb falls within the origin of replication of the pUC plasmid backbone. The SIDD profile for construct pEP-IR also shows the strongly destabilized areas of the IR element.

Constructs were made by introducing modifications in the prototype vector pEPI-eGFP, which served as control in all experiments (Fig. 1a). We used promoter EF1/HTLV which has been shown to support homogeneous, high expression of the reporter gene eGFP^31^, and promoter SFFV which has been shown to enhance the long-term expression of a transgene in primary human haematopoietic cells *in vivo*^32^. Specifically, we replaced the pCMV in the transcription cassette CMV-eGFP-S/MAR of plasmid pEPI-eGFP, with the EF1/HTLV promoter to construct plasmid pEPI-EF1/HTLV, and with the SFFV promoter to construct plasmid pEPI-SFFV. Subsequently, the β-globin Replicator was added to the pEPI-SFFV plasmid (Fig. 1a), to construct plasmid pEP-IR. As shown below, the performance of vectors pEPI-SFFV and pEPI-EF1/HTLV was found to be comparable and therefore, only one plasmid, the pEPI-SFFV, was used for the insertion and analysis of the role of the IR element.

We performed a Stress Induced Duplex Destabilization (SIDD) profile analysis of plasmids’ DNA (Fig. 1b), in order to determine the distribution of the strand separation potential along the various sites in the DNA of each plasmid constructed^33^. Strand separation is an important contributor to the two major DNA functions, namely transcription and replication. Any change along the plasmid sequence may result in an energetic transition, leading to redistribution of the strand separation potential among the different parts of the circular plasmid DNA^33^, which, in turn, may affect the efficiency of transcription and/or replication of the plasmid. SIDD analysis here served the purpose of investigating the possibility that replacement of pCMV by other promoters, or the insertion of the β-globin Replicator, may introduce drastic changes in the distribution of destabilization potential along the DNA of the respective plasmid, as compared to the control plasmid. Our analysis shows that no changes in the SIDD profiles of backbone and promoter sequences are brought about by the promoter replacements (Fig. 1b). Additionally, the β-globin Replicator site presents a new position of high destabilization potential in the DNA of plasmid pEP-IR and, according to our previous data^27^, this may indicate higher capacity for the vector’s retention in the cell nucleus.

### Transfections in K562 cells

We performed gene transfer experiments into cells of the haemopoietic cell line K562, in order to investigate the ability of the novel vectors to establish stably transfected cell lines, as episomes. The percentage of fluorescent cells containing one of the plasmids pEPI-eGFP, pEPI-EF1/HTLV, pEPI-SFFV and pEP-IR was determined 48 hours after transfection, by flow cytometry, in, at least, three transfections and was found to be on average, 26.7 ± 7.3%, 27.3 ± 3.1%, 27.8 ± 8% and 44.5 ± 8.5%, respectively (see Suppl Fig. S1). G418 was applied 48 hours post-transfection, and stably transfected cell lines were obtained 7 to 10 days later. Cells in stably transfected cultures were eGFP^+^ for 5 months of continuous culture, with and without selection. Cells growing without selection were tested for resistance to G418 at 3 and 5 months of culture and were found to be resistant to the antibiotic, while untransfected K562 cells died 4–5 days after the addition of G418 (see Suppl Fig. S2). Results show that all three novel vectors are functional in establishing stable transfection in K562 cells, as does the control. Establishment in K562 cells of vectors pEPI-eGFP, pEPI-EF1/HTLV and pEPI-SFFV is in the area of 4% to 5% (data not shown) and that of vector pEP-IR was found (see Methods) to be around 8 ± 2.3%.

**Figure 2:**
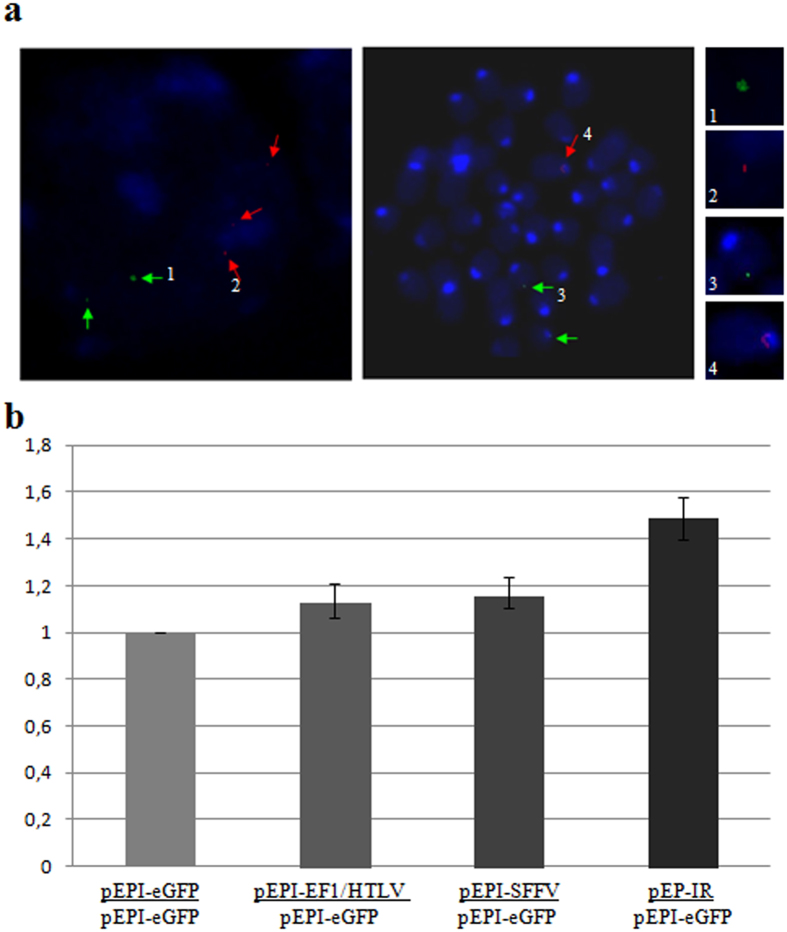
Episomal status of vector pEP-IR and expression of thee eGFP transgene in transfected K562 cells. (**a**) K562 cells transfeced with vector pEP-IR, after three month of continuous culture supplemented by G418 were analysed by fluorescent *in situ* hybridization (FISH). Representative interphase (left) and metaphase (middle) spreads are shown. Red arrowheads show the pEP-IR plasmid as non-integrated episomes and green arrowheads show the control HBB loci in up to three copies of chromosome 11. Selected points 1, 2, 3 and 4 are shown in enlarged photos to the right. (**b**) eGFP expression in K562 cells transfected with pEPI-eGFP, pEPI-EF1/HTLV, pEPI-SFFV and pEP-IR plasmids within the 5-months period of continuous culture, as estimated by RT-qPCR. Values were normalized against pEPI-eGFP, which was taken as 1.

The possibility of integration of plasmid pEP-IR into the endogenous genetic material of the K562 cells was investigated by Fluorescent *In Situ* Hybridization (FISH) (Fig. 2a). No integration events were detected in about one hundred metaphase cells examined. It is deduced that expression of eGFP in this study derives solely from episomal vector pEPI-IR.

Expression of eGFP in transfected K562 cells was estimated at the mRNA level by RT-qPCR (Fig. 2b). Expression of eGFP for each vector was found to be steadily maintained throughout the period of 5 months of culture, and Fig. 2b depicts data at the end of 5 months. Similar results were obtained at the protein level by the use of flow cytometry to estimate the mean fluorescence intensity 3 and 5 months post-transfection (see Suppl Fig. S3). Analysis of transgene expression in K562 cells, transfected with the novel vectors, verified consistent functionality for all vectors.

### Transfections in CD34^+^ cells

Transfections in CD34^+^ haematopoietic progenitor cells were carried out to study the various aspects of the vectors’ performance in these cells.

### All three novel vectors transfect CD34^+^ cells efficiently and stably

**Figure 3:**
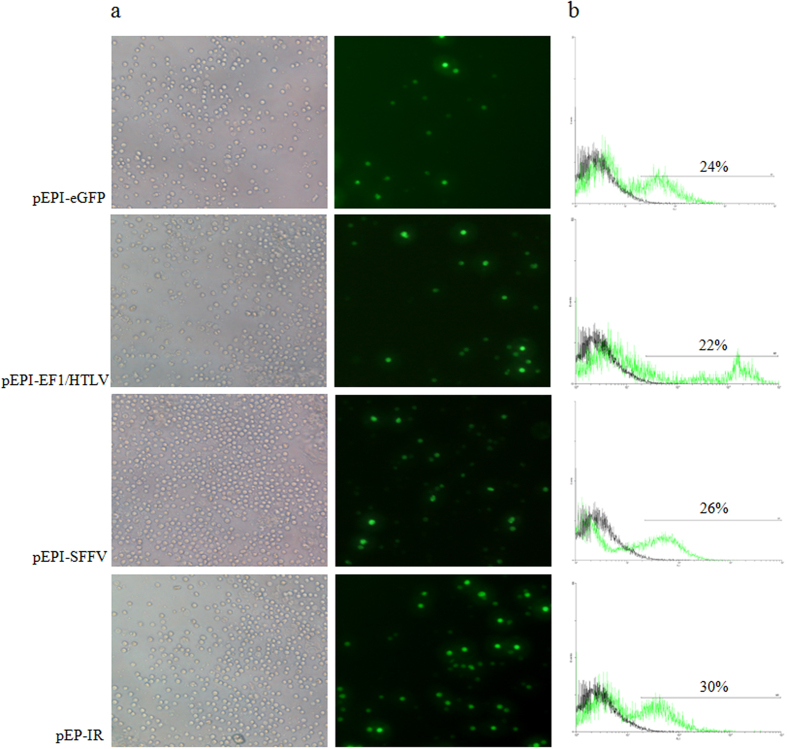
Documentation of eGFP expression in transfected CD34^+^ cells. eGFP expression was documented in CD34^+^ cells carrying the control pEPI-eGFP vector and each of the three experimental vectors respectively, 48 hours after transfection. Results are shown (**a**) from phase contrast (left column) and fluorescent microscopy (right column) and (**b**) from flow cytometry, giving the estimated percentage of fluorescent, transfected cells, for each vector used. y-axis records counts of cells and x-axis records FL1-eGFP fluorescence.

DNA of plasmids pEP-IR, pEPI-SFFV, pEPI-EF1/HTLV and of control plasmid pEPI-eGFP were used to transfect CD34^+^ cells, isolated from leukapheresis products. Transfection was carried out by nucleofection and viability, estimated after 24 hours by trypan-blue staining, was 60 ± 6%, while that of the untransfected CD34^+^ cells of the same age was 94 ± 2%. The percentage of eGFP^+^ cells from at least three independent transfections, as determined by flow cytometry 48 hours post-transfection, was 24.7 ± 4% for control vector pEPI-eGFP, 22.9 ± 2% for vector pEPI-EF1/HTLV, 26.8 ± 2% for vector pEPI-SFFV and 31.8 ± 3% for vector pEP-IR (Fig. 3). No antibiotic selection was applied in transfected CD34^+^ cell cultures.

Transfected CD34^+^ cells were sorted by fluorescence-activated cell sorting (FACS) and eGFP^+^ cells were placed in colony-forming cell culture (CFC) in order to investigate a) whether the plasmids are retained and to what extend and b) whether they maintain expression during erythroid differentiation of HSCs.

**Figure 4:**
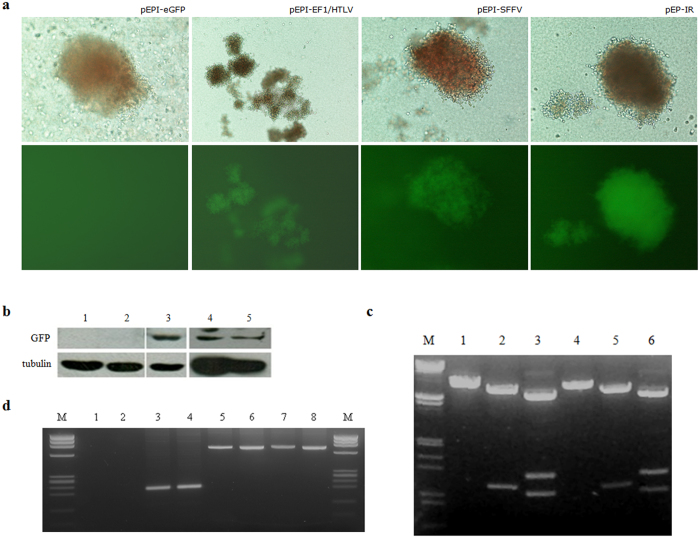
(**a**) Colonies derived from CFC assays in semisolid cultures (14 days) of FACS-sorted CD34^+^/eGFP^+^ transfected CD34^+^ cells. Colonies of cells containing the control plasmid pEPI-eGFP or one of the three experimental plasmids are shown. Phase contrast microscopy (upper row) and fluorescent microscopy (lower row) reveal that cell colonies with each of the three experimental vectors express eGFP, in contrast to cell colonies with the control plasmid pEP-eGFP, which lack eGFP expression and thus detectable fluorescence. (**b**) Western blot analysis of protein preparations from CFC culture colonies, derived from transfected CD34^+^ cells. 1. untransfected cells; 2. cells transfected with pEPI-eGFP; 3. cells transfected with pEP-IR; 4. cells transfected with pEPI-SFFV; 5. cells transfected with pEPI-EF1/HTLV (see Suppl Fig. S5). (**c**) Plasmid rescue experiment with restriction analysis of pEP-IR vector DNA used as input for transfections (lanes 1, 2, 3) and DNA prepared from a colony of *E. coli* transformed with a HIRT extract (see Methods for details) of pEP-IR rescued plasmid; Substrates (and restriction digests) are as follows. M: λ DNA (*HindIII*/*EcoRI*); 1. pEP-IR (*AseI*); 2. pEP-IR (*XmnI*); 3. pEP-IR (*AvrII*); 4. plasmid rescued (*AseI*); 5. plasmid rescued (*XmnI*); 6. plasmid rescued (*AvrII*). (**d**) Colony PCR of DNA extracts from CFC culture colonies (14 days) derived from FACS-sorted CD34^+^/eGFP^+^ transfected CD34^+^ cells. M. Φχ174RF DNA/*HaeIII* Digest Marker (HT Biotechnology LTD); eGFP^+^ and eGFP^−^ indicates fluorescent and non-fluorescent CFC culture colonies derived from transfected CD34^+^ cells respectively. 1. eGFP^−^-cell-colonies transfected with pEPI-EF1/HTLV; 2. eGFP^−^-cell-colonies transfected with pEPI-SFFV; 3. eGFP^+^-cell-colonies transfected with pEPI-EF1/HTLV; 4. eGFP^+^-cell-colonies transfected with pEPI-SFFV; 5. eGFP^−^-cell-colonies transfected with pEPI-SFFV; 6. eGFP^−^-cell-colonies transfected with pEPI-SFFV; 7. eGFP^+^-cell-colonies transfected with pEPI-EF1/HTLV; 8. eGFP^+^-cell-colonies transfected with pEPI-SFFV. Runs 1, 2, 3 and 4 refer to PCR reactions with eGFP primers (amplified fragment size 232 bp) and 5, 6, 7 and 8 to PCR control reactions with β-globin primers (amplified fragment size 713 bp).

We carried out transfections (4 to 5) with each of the three novel vectors and the control vector in parallel, under the same conditions and with the same pool of cells. We used, on average, 1000 eGFP^+^/CD34^+^ FACS sorted cells per dish placed in CFC assay. Fourteen days later, all colonies deriving from CD34^+^ cells transfected with the control vector pEPI-eGFP were eGFP^−^ in fluorescence microscopy, similarly to previous experiments with cord blood CD34^+^ cells^19^. In sharp contrast, intense fluorescence was observed in colonies produced from cell cultures carrying vectors pEPI-EF1/HTLV, pEPI-SFFV and pEP-IR (Fig. 4a). The colonies obtained were, on average, 115.8 for pEPI-eGFP, 100.3 for pEPI-EF1/HTLV, 98 for pEPI-SFFV and 154 for pEP-IR (Table 1). The fluorescent colonies obtained in each case were 0%, 49.8%, 56.9% and 100% respectively. These data reveal two new findings: firstly, the replacement of CMV promoter by either EF1/HTLV or SFFV promoter has a positive effect on eGFP expression in CD34^+^ cells and the presence of fluorescent colonies represent cells with efficient plasmid retention, leading to establishment; secondly, the presence of IR has a catalytic effect on further increase in plasmid retention, as, virtually, every colony developing from cells transfected with vector pEP-IR is fluorescent. Importantly, the differences in colony numbers referring to vector pEP-IR versus vector pEPI-eGFP are statistically significant (p = 0.0008). This was not documented for any other pair of vectors.

These data allow the evaluation of the rates of establishment of the novel vectors in CD34^+^ cells, on the basis of reported, similar gene transfer in CHO cells^34^, given by the ratio of colonies appearing after 14 days of culture over transfected, seeded cells. Here, the ratio of fluorescent, stably transformed colonies over eGFP^+^/CD34^+^ seeded cells after 14 days of the CFC assay (16 days after transfection) is represented by the percentages 0% for pEPI-eGFP, 5%, for pEPI-EF1/HTLV, 5.5% for pEPI-SFFV and 15% for pEP-IR. These values show that the relative capacity for establishment is comparable between vectors pEPI-EF1/HTLV and pEPI-SFFV as well as between them and the respective values for the control plasmid pEPI-eGFP in permissive conditions, while for vector pEP-IR it is increased 3-fold, compared to that of the other two vectors.

Noticeably, the CFC data obtained here allow for the definition of the establishment rates of the respective vectors in HSCs, presenting a platform for comparison amongst them, but they do not provide a proof for long-term establishment of the vector plasmids in HSCs, for which long-term culture studies are needed.

The data reported in Table 1 also reveal an unexpected finding, namely that, in the case of CFU-GEMM, the fraction of number of (non-fluorescent) colonies over total colonies for pEPI-eGFP (39/463) appear to be significantly higher than those of vectors pEPI-SFFV (0/490) and pEP-IR (0/770) (p = 0.0077). It is possible that the vector pEPI-eGFP supports CFU-GEMM survival or, alternatively, the derived vectors have a detrimental effect on it, but no further experiments were carried out on this issue.

### The novel vectors support transgene expression and are maintained as free plasmids in the progeny of CD34^+^ cells

The presence of fluorescent cell colonies under fluorescent microscopy is a strong indication of the expression of the transgene eGFP in CD34^+^ cells, transfected by each of the three novel vectors. As the determination of eGFP expression is crucial in this work, we confirmed the above observation by Western blot analysis of cell lysates, prepared from fluorescent colonies only, with an anti-eGFP antibody (Fig. 4b). eGFP was clearly detectable in preparations derived from cells transfected with vectors pEPI-SFFV or pEP-IR, in two different experiments, but not in preparations from cells transfected with the control vector pEPI-eGFP.

To test whether these vectors were actually maintained as free plasmids in the transfected cultures, we performed a plasmid rescue assay with DNA isolated from eGFP positive cell colonies (Fig. 4c). Results show that the restriction pattern of the DNA from rescued plasmids is the same as that of input plasmid DNA, used for the transfection of the CD34^+^ cells, and this demonstrates that vectors exist as free, non- integrated plasmids.

### Only fluorescent cell colonies deriving from transfected CD34^+^ cells carry vectors

As shown in Table 1% of colonies carrying plasmid pEPI-EF1/HTLV and 43.1% of colonies carrying plasmid pEPI-SFFV were non- fluorescent. To test whether the vector is absent or present but silenced as a result of epigenetic alterations in these cells, we performed PCR for the eGFP gene in DNA from fluorescent and non-fluorescent colonies, both deriving from eGFP positive, CD34^+^ FACS-sorted cells, transfected with vector pEPI-SFFV (Fig. 4d). Single, non-fluorescent-colony PCR^19^ applied to about 10 colonies, was negative for plasmid retention. Extended analysis was carried out with DNA from at least 90 pooled, non-fluorescent colonies in each PCR, so as to maximize chances of plasmid detection in the case retention is a rare event and PCR using equal number of fluorescent colonies were carried out, as control. The data showed that non-fluorescent colonies were negative for the presence of the transgene, while control PCR was positive for the same gene. To determine the detection threshold of this assay, PCRs using DNA from the 90 pooled, fluorescent colonies in dilutions 1/100, 1/150, 1/250, 1/300 and 1/500 were performed. Plasmid presence was documented for dilutions up to 1/250, while results for the remaining dilutions were negative (data not shown). The data document that non-fluorescent colonies do not carry plasmid DNA. Evidently, the non-fluorescent colonies of cells that are devoid of vectors derive from transfected, eGFP^+^ cells, as a result of inefficient mitotic segregation of plasmids and plasmid loss during culture.

### Plasmid copy number per fluorescent cell in the progeny of transfected CD34^+^ cells

The determination of the plasmid copy number per cell provide a measure of the vector potential and is necessary for estimating the transgene expression per plasmid copy. For each novel vector in this study the plasmid copy number per cell was determined, by qPCR for Absolute Quantification (see Methods) using eGFP-specific primers and input DNA from whole fluorescent colonies. Plasmid copy number per cell was found to be 0.58 for vector pEPI-EF1/HTLV, 0.69 for vector pEPI-SFFV and 1.78 for vector pEP-IR.

The estimations for vectors pEPI-EF1/HTLV and pEPI-SFFV show that the respective fluorescent colonies include a high number of cells that do not contain vector plasmid. This is also documented by fluorescent microscopy, in which fluorescent colonies for these two vectors are mixed, containing fluorescent as well as non-fluorescent cells (Fig. 4a). Taking into account that only fluorescent cells carry plasmids, the plasmid copy number for each vector should be determined per *fluorescent cell,* provided that one can estimate the percentage of fluorescent cells in mixed colonies.

**Figure 5:**
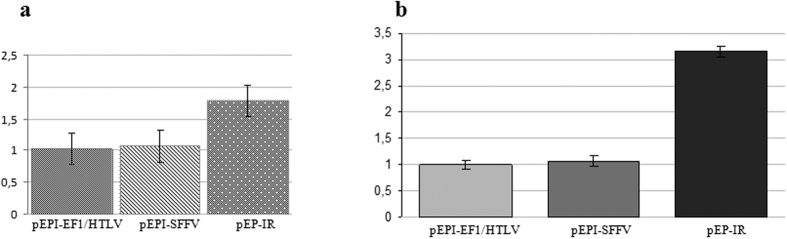
(**a**) Plasmid copy number per eGFP^+^ cell from CFC culture colonies derived from FACS-sorted CD34^+^/eGFP^+^ cells transfected with pEPI-EF1/HTLV, pEPI-SFFV or pEP-IR, was estimated by qPCR and normalized as explained in Results. (**b**) eGFP expression per plasmid copy number per fluorescent (eGFP^+^) cell is shown. eGFP expression in mRNA from colonies derived from FACS-sorted CD34^+^/eGFP^+^-transfected CD34^+^ cells with pEPI-EF1/HTLV, pEPI-SFFV and pEP-IR. eGFP expression was estimated with RT-qPCR quantification analysis and normalized with the copy number of each plasmid per fluorescent (eGFP^+^) cell (explanations in Results).

We assumed that, for each vector (pEPI-EF1/HTLV or pEPI-SFFV) -cell culture, the probability of plasmid loss should be the same among mitoses occurring at any time in transfected CD34^+^-derived/eGFP^+^, FACS-sorted cells. In particular, the likelihood of plasmid loss is not expected to be different between mitoses in the original seeded cells that produced fluorescent and non-fluorescent **colonies** on one hand and mitoses within a growing, mixed colony that produced fluorescent and non-fluorescent **cells** on the other hand. We can, therefore, consider that within a mixed colony, the percentage of fluorescent cells over total number of cells is equal to the percentage of mixed colonies over total number of colonies (mixed and non-fluorescent) that is given for the respective vector-cell culture. Subsequently, we carried out normalization of the plasmid copy number per cell that was obtained by absolute quantification, taking into account the respective percentage of fluorescent colonies for each vector, namely 49.8% for vector pEPI-EF1/HTLV and 56.9% for vector pEPI-SFFV (Table 1). This gave the values 1.16 plasmid copy number per *fluorescent cell* for vector pEPI-EF1/HTLV and 1.21 plasmid copy number per *fluorescent cell* for vector pEPI-SFFV (Fig. 5a).

For vector pEP-IR the plasmid copy number per cell was determined as 1.78 (Fig. 5a). This is also the plasmid copy number per *fluorescent cell*, as 100% of colonies are fluorescent. These data are in accordance with the data from fluorescent microscopy, in which not only all colonies but all cells within a colony, as far as it can be determined by observation, are fluorescent. Therefore, the presence of IR in the plasmid pEP-IR results in an increase in plasmid copy number per *fluorescent cell* by 50%.

### Relative capacity of novel vectors for eGFP expression in the progeny of transfected CD34^+^ cells

The eGFP mRNA per plasmid copy was determined for each of the three novel vectors by RT-qPCR, taking into account the plasmid copy number per *fluorescent cell* (described in the previous section), derived from CD34^+^ cells that were transfected accordingly. The *relative* abundance of each mRNA was estimated considering the value of eGFP mRNA per plasmid copy for vector pEPI-EF1/HTLV as one (1) and by calculating the respective values for vectors pEPI-SFFV and pEP-IR, each as a fraction over that of vector pEPI-EF1/HTLV (Fig. 5b). Thus, it is shown that cells carrying either vector pEPI-EF1/HTLV or vector pEPI-SFFV have almost the same capacity for eGFP expression per plasmid copy. Significantly, cells carrying vector pEP-IR are capable of 3-fold higher eGFP expression per plasmid copy, than cells carrying either of the two vectors that do not carry the IR. It is possible that the presence of the IR element has an enhancing effect on nearby eGFP transcription, resulting in triplication of steady-state mRNA level of the transgene.

Noticeably, fluorescence in the cell-colonies carrying vector pEP-IR is brighter and more homogeneous, compared to the colonies derived from cells transfected with either of the other two experimental vectors (Fig. 4a).

### Transfer of the vector pEP-IR in thalassaemic mouse, haematopoietic, progenitor cells

We transferred vector pEP-IR into haematopoietic progenitor cells of the Hbb^th-3^ mouse model which resembles human thalassaemia intermedia^35^ for the sole purpose of investigating whether or not it can be delivered and it is capable of supporting trangene expression in these cells.

Whole bone marrow cells from the thalasseamic Hbb^th-3^ mouse as well as from wild type C57Bl6 mouse were transfected with pEP-IR vector and 24 hours later cells were analyzed by flow cytometry and transfection efficiency was comparable, from 7% to 9%. Cells were stained as described in Methods and results show that viability of the transfected, thalassaemic cells was 18%, while that of the control, untransfected, thalassaemic cells was 68%. eGFP^+^ cells were found only in the transfected, thalassaemic cell cultures and were 2.5% of the live cells. Amongst eGFP^+^ cells, 0.4% were Lin^−^/Sca^+^ cells (characterizing haematopoietic progenitor cells) (see Suppl Fig. S4). It is shown that vector pEP-IR can achieve some uptake in haematopoietic progenitor cells, derived from a thalassaemic mouse model, but at extremely low rate. Importantly, it can support transgene expression in these cells.

## Discussion

In this work, we used episomal vectors pEPI-EF1/HTLV, pEPI-SFFV and pEP-IR and control vector pEPI-eGFP to investigate vector establishment, transgene expression and other aspects of episomal gene transfer in K562 and haematopoietic progenitor cells:

1. Transfections of K562 cells determined the functionality of vectors pEPI-EF1/HTLV, pEPI-SFFV and pEP-IR, as they produced stably transfected cell lines, just like the ones obtained with the control plasmid pEPI-eGFP. This was also expected by the vectors’ SIDD profiles, which maintain the same site of strong destabilization potential corresponding to the S/MAR element, despite the promoter exchange and the addition of the IR. The presence of IR element in vector pEP-IR enhances vector’s establishment and eGFP expression in K562 cells, but to a lesser degree than in the progeny of CD34^+^ cells (Figs 2b and 5b). The haematopoietic K562 cell line is a somewhat abnormal cellular system, in that it presents karyotype inconsistency in long-term culture, but nevertheless, a tendency for higher eGFP expression in the presence of IR in K562 is clear.
2. Vector pEP-IR is delivered at extremely low efficiency, but supports transgene expression in mouse haematopoietic progenitor cells.
3. Finally, transfections of CD34^+^ human haematopoietic progenitor cells revealed that the β-globin Replicator greatly enhances all main properties of this S/MAR-based episomal vector. The specific features of vector pEP-IR with regard to transfections into CD34^+^ haematopoietic progenitor cells are presented below.

### The novel vectors are capable of mediating stable transfection of CD34^+^ cells

Although all vectors transfect CD34^+^ cells with satisfactory rates, only the three novel vectors pEPI-EF1/HTLV, pEPI-SFFV and pEP-IR, but not the control vector pEPI-eGFP, give fluorescent colonies in the progeny of CD34^+^ cells, over the two-week time period of the CFC assay (Fig. 3, Table 1) and actually, the number of fluorescent colonies concerning the vector pEP-IR is statistically significantly higher versus the control vector pEPI-eGFP. The data with control vector pEPI-eGFP are similar to our previous ones, in that the vector is perfectly functional in a number of established cell lines but not in CD34^+^ cells. It seems that pCMV, driving eGFP transcription through the S/MAR, is silenced to non-functionality in transfected CD34^+^ cells, whether these derive from cord blood, as in our previous study, or from adult bone marrow, as in this study, most probably by epigenetic mechanisms. To the contrary, promoters EF1/HTLV and SFFV are competent in supporting sustained transcription through eGFP-S/MAR in CD34^+^ cells, as well as in the differentiated cells produced in CFC assay, thus providing the necessary condition for the S/MAR to facilitate nuclear retention of episome(s) in these cells. Evidently, conclusions on pCMV function in K562 cells, as in this setting, cannot be extrapolated to primary cell cultures.

Furthermore, the CFC analyses for total CFU-GEMM numbers raised the possibility that replacement of the pEPI-eGFP promoter may interfere with survival of more rudimentary HSCs. While this does not affect in any way the essence of this work, nevertheless, possible confirmation of this finding may require specific interventions e.g. in vector structure or culture conditions, for future application in HSCs.

### The β-globin Replicator has a positive effect on vector rate of establishment in CD34^+^ cells

The estimated ratios of fluorescent, stably transfected colonies to transfected, seeded cells are 5% for pEPI-EF1/HTLV, 5.5% for pEPI-SFFV and 15% for pEP-IR, documenting that the relative capacity of vectors pEPI-EF1/HTLV and pEPI-SFFV for establishment is virtually the same, while for vector pEP-IR it is 3-fold higher.

These estimates can be of clinical importance, particularly in the case of vector pEP-IR, which, in each of a series of seven transfections of CD34^+^ cells routinely produced 100% fluorescent cells. Practically, vector pEP-IR is expected to persist in all cells that give rise to the differentiated cell-progeny of CD34^+^ cells, that is in 10% to 20% of the total sorted CD34^+^/eGFP^+^ cells.

Furthermore, we have shown that vector pEP-IR is capable of ensuring 50% increase in copy number per *fluorescent cell*, compared to the parent vector pEPI-SFFV (Fig. 5a).

These data establish that the β-globin Replicator exerts a definite positive effect on vector establishment rate and the number of vector copies in the nucleus of the CD34^+^ haematopoietic progenitor cells and their differentiated progeny.

### The β-globin Replicator has a strong positive effect on eGFP expression in the progeny of CD34^+^ cells

Vector pEP-IR ensures three times the eGFP mRNA per plasmid copy compared to vectors pEPI-EF1/HTLV and pEPI-SFFV (Fig. 5b). This is a novel information concerning IR function within an episomal vector. It is not obvious how enhancing plasmid DNA replication *per se* can increase transcription of eGFP, but, considering that the IR is positioned immediately upstream the proximal SFFV promoter, a possible replicative activity at the IR site may bring about a higher degree of DNA destabilization and of open chromatin state at this site, which, in turn, may allow a higher degree of transcriptional activity at the SFFV promoter. So far, the IR has only been used within S/MAR-based vectors. Our data from previous work^27^ have shown that the existence of this particular combination of high destabilization potential sites, S/MAR and IR, in an episome derived from plasmid pCEP4, is associated with high efficiency of nuclear retention of the plasmid in Jurcat cells. It is possible that the IR operates in synergy with the S/MAR element, as it has been shown that the coexistence of a MAR element and the β-globin Rep-P replicator, i.e., the AT-rich region, the MAR-like element, and the AG-rich region, is required for extrachromosomal amplification^36^.

In vector pEP-IR the IR is located next to the 5′ side of the SFFV promoter, a position relatively far from the S/MAR, while from our previous work^27^ we know that the IR can operate when placed 3′ downstream the poly-A signal of the transgene transcription cassette, a position close to the S/MAR.

On the whole, it is documented that the IR can contribute to plasmid persistence (1) from two different plasmid backbones, pCEP4 and pEPI-1, (2) in established cell lines as well as in cultures of primary cells, and (3) from two different positions within the vector DNA sequence in relation to proximity to S/MAR element.

The IR may exert these effects as a *bona fide* mammalian replication element, but alternative or complementary mechanisms may contribute to its function. The IR element includes DNA sequences of the β-globin promoter, with binding sites for a number of ubiquitous and tissue-specific transcription factors. Additionally, the IR bears a predicted, consensus binding site for the DNA-binding protein CTCF (CCCTC-binding factor), a well-known, ubiquitously expressed nuclear protein, highly conserved from Drosophila to mouse and man. CTCF is prominently involved in the three-dimensional organization of chromatin and a key player in transcriptional regulation in vertebrates^37^, while it appears to possess strong insulating activity *in vitro*^38^. Importantly, it has been found to confer episomal stability to herpesvirus genomes in latently infected human T cells^38^.

### Transfection of murine HSCs

Vector pEP-IR is capable of achieving delivery in murine HSCs, but to an extremely low level. On the other hand, vector pEP-IR is capable of supporting transgene expression in these cells, which may prove to be important in pre-clinical studies.

### Towards pEP-IR in preclinical studies

Successful transfections in murine HSCs are of critical importance for pre-clinical, *in vivo* studies of episomal gene transfer, and non-delivery is a major problem, particularly for plasmids of long DNA sequence, like pEP-IR. Thus, our system is restricted by what is known as a major experimental shortcoming for episomal gene transfer, namely, the lack of sufficient plasmid delivery into cells, in this case murine progenitor cells. A somewhat similar situation is also encountered with transfections into the human CD34^+^ cells, as the respective transfection efficiency is adequate for current experimentation, but most probably will not be sufficient for *in vivo* studies involving the transfer of transfected CD34^+^ cells into appropriate mouse disease model. To address this issue, the development of a non-integrating lenti-viral vector^5^ engineered for the delivery of episomes, could offer a valued solution. Additionally, work on the β-globin Replicator and, in general, on the pEP-IR DNA topology, may lead to a more confined sequence for this element’s function, and to shorter plasmid DNA, which, on the whole, may be more suitable for gene therapy applications.

### Conclusions

It is shown that promoters SFFV and EF/HTLV are efficient in driving transgene and S/MAR transcription in episomes derived from pEPI-eGFP and that the respective vectors can be maintained in CD34^+^ cells and their progeny in CFC assays. Additionally, it is documented, for the first time, that the presence of the IR in S/MAR-based vectors for gene transfer in CD34^+^ human, haematopoietic progenitor cells, has a definitive, enhancing effect on episomal vector performance, namely the rate of establishment, vector copy number per cell and transgene expression per plasmid copy. The present study also points to the need of addressing the delivery issue for episomes.

The IR element is thus emerging as one more chromosomal element along with other such elements^39^,^40^, like the extensively studied S/MAR, the ubiquitous chromatin opening elements (UCOEs) and the cHS4 insulator, which contribute to the formation of autonomously replicating genetic elements. Such autonomous replicons are considered to be suitable for therapeutic and biotechnological applications as well as for the study of the compartmentalization of the nucleus.

## Methods

### Human and animal cells

Informed consent was obtained from all CD34^+^ cell donors. CD34^+^ cells from adults were isolated from leukapheresis products, collected from G-CSF-mobilized healthy donors in accordance with the relevant guidelines of the Bone Marrow Transplantation Unit, and all experimental protocols were approved by the Ethics Committee of the Medical School University of Patras (Nr. 525/07.09.06).

All animal experiments were approved by the Animal Care and Use Committee of the Veterinary Directorate, Prefecture of Thessaloniki, Region of Central Macedonia, Greece and performed in accordance with relevant guidelines and regulation.

### Plasmid construction

Plasmids pEPI-EF1/HTLV (6.6 kb) and pEPI-SFFV (6.5 kb) (Fig. 1a) were constructed by deleting the CMV promoter (0.7 kb) from plasmid pEPI-eGFP (6.7 kb) after DNA digestion with the restriction enzymes *NheI* and *PciI* in the first case and *AgeI* and *PciI* in the second case, resulting in linear vectors with sticky ends. Promoters EF1/HTLV and SFFV were PCR amplified from plasmids pBC-EF1/HTLV and SFFV-pRRL, respectively. The PCR products EF1/HTLV (0.6 kb) and SFFV (0.5 kb) were also restricted with the corresponding enzymes above and were inserted into the respective linearized plasmid vector with the use of DNA Ligation Kit (Takara) according to the manufacturer’s instructions. Plasmid pEP-IR (8.2 kb) (Fig. 1a) was constructed by digestion of the pEPI-SFFV (6.5 kb) with the *NheI* and *PciI* restriction enzymes, and the linear vector was ligated to the IR (1.6 kb) PCR fragment digested also with *NheI* and *PciI.*

### Cloning PCR

PCR reactions were performed using 0. 25 u Taq polymerase (Invitrgen), 0.5 pmol dNTPs (Fermentas) and 0.25 pmol of each specific primer. All primers contained the restriction site required for subcloning, underlined in the primer sequence below. Sequence fidelity of clones was confirmed by DNA sequencing (outsourced).

For the EF1/HTLV promoter amplification: *PciI*_Forward: 5′-CCA CAT GTC ATC AAA ACA AAA CGA AAC AAA AC-3′ and *NheI*_Reverse: 5′-CAG CTA GCC AAG GTT AG TAG TCG ACG TGT CC-3′. For the SFFV promoter amplification: *PciI*_Forward: 5′-CCA CAT GTA TGA AAG ACC CCA CCT GTA GGT TTG G-3′ and *AgeI*_Reverse: 5′-CAC CGG TCG GGT ACCCGG GCG ACT C-3′. For the IR fragment amplification: *PciI*_Forward: 5′-CGA CAT GTG CTC GGA TCC ACT AGT A-3′and *NheI*_Reverse: 5′-CAG GCC GCT AGC CGC CAG TGT GAT GGA TA-3′. PCR conditions were: denaturation at 95 °C for 10 min followed by 35 cycles (95 °C for 1 min, 55 °C for EF1/HTLV and SFFV promoter and 60 °C for IR, for 0.60 min, 72 °C for 90 sec) and a final extinction 72 °C for 10 min.

### CD34^+^ Cell isolation

CD34^+^ cells were isolated from low-density mononuclear cells (MNC) derived from peripheral blood of initiated healthy donors, using gradient isolation (1.077 g/ml Ficoll-Paque, Biochrom) and immunomagnetic selection, using a combination of the Miltenyi CD34 MultiSort kit (Miltenyi Biotec) and the LS Columns (Miltenyi Biotec) in accordance with the manufacturer’s instructions. In all preparations, CD34^+^ cell purity exceeded 98%, as estimated by flow cytometry (data not shown).

### Cell culture and transfection

K562 cells were cultured in DMEM (Gibco) supplemented with 10% FCS, 2 mM L-glutamine, 50 u/ml penicillin and 50 mg/ml streptomycin. All reagents were purchased from Invitrogen.

CD34^+^ cells were cultured at a concentration of 2 to 5 × 10^5^ cells/ml in IMDM with 10% FCS supplemented with the recombinant human (rh) cytokines: 20 ng/ml rh stem cell factor (SCF), 50 ng/ml rh thrombopoietin (TPO) and 50 ng/ml rh Flt-3 ligand (FL) (complete, serum-free medium). Cell culture reagents were purchased from Invitrogen and recombinant cytokines were purchased from BioSource.

K562 cells were transfected by electroporation in a GenePulserII electroporator (BioRad). Cell suspension 2 × 10^6^ up to a final volume of 500 μl was mixed with 15 μg of plasmid DNA, was placed in a 0.4 cm electroporation cuvette (BioRad,) and electroporated at 220 V, 975 mF (t: 23.2–25.1). After electroporation, cells were immediately transferred to 10 ml preheated, complete medium and incubated at 37 °C in 5% CO_2_. Transfected cells were selected 48 hours after transfection by the addition of Geneticin (G418) (Invitrogen) in the medium at an initial concentration of 800 mg/ml. 14–19 days later, stably transfected, selected cells were split in two and cultured further, either with G418 (at a concentration of 400 mg/ml) or without G418.

CD34^+^ cells were transfected with nucleofection using the Amaxa NucleofectorII (programme U008) with Human CD34^+^ Cells Nucleofector solution following the company’s instructions.

### Fluorescence Microscopy analysis, Flow Cytometric Analysis and Cell Sorting

eGFP expression of the transfected cells was examined and documented under a fluorescent microscope (Nikon Eclipse TE 2000). The filter cube used in the microscope was the fluorescein isothiocyanate dichroic filter set (excitation at 450–490 nm; emission at 520 nm). The software used for analysis was FlowJo 7.6 software (Tree Star, Ashland, OR).

Approximately 3–5 × 10^5^ cells were washed twice with 1x PBS and analyzed on an EPICS-XL flow cytometer (Coulter).

Approximately 5 × 10^5^ cells were washed and incubated with an anti-CD34-phycoerythrin (PE)-conjugated monoclonal antibody (Pharmingen) for 30 min at 4 °C. Dead cells were excluded by gating based on propidium iodide (PI) (Pharmingen). Transfected cells were sorted with a FACS Vantage (BD) as previously described^19^.

### Estimation of vector pEP-IR % establishment in K562 cells

Two parallel transfections were performed and GFP^+^ cells were FACS sorted after 24 h. Cultures of known number of these GFP^+^ cells were set up and flow cytometry was applied for the recording of percentage of surviving eGFP^+^ cells in consecutive days, taking into account the cell death for each day. This percentage was falling steadily until day 7 or 8, when it started increasing again, giving rise to a full, stably transfected culture one week later. The percentage of GFP^+^ cells on days 7 or 8 provides the rate of establishment in K562 cells.

### Colony-forming cell (CFC) Assay

Selected CD34^+^ cells expressing eGFP (1 × 10^3^ cells/ml) were plated in methylcellulose medium supplemented with cytokines Metho-Cult GF + H4435 (Stem Cell Technologies). After 10–14 days incubation at 37 °C in 5% CO_2_, colonies were counted on an inverted fluorescence microscope (Nikon Eclipse TE 2000). Single colonies, after 14 days incubation, were randomly collected, pooled and used for DNA, RNA and protein extraction (AllPrep DNA/RNA/Protein Mini Kit, Qiagen).

### Calculation of stress-induced destabilization profiles

The analysis of the SIDD profile was performed as described previously^27^, using the WebSIDD internet application^41^. By this method two parameters are evaluated: the equilibrium probability (p) of denaturation at single base pair (x) resolution and the energy G(x), also called destabilization energy, which is the incremental free energy required for the denaturation of the base pair at position x. The calculation of G(x) makes possible the detection of fractionally destabilized sites -locations that have a high probability of denaturation- and duplex opening can be driven by relatively small incremental energy. The data set produced is an approximate statistical mechanical equilibrium distribution of a population of identical molecules among its accessible states, using structural and energy parameters that have been verified experimentally.

### DNA fluorescence *in situ* hybridisation (FISH)

K562 cells stably transfected with the pEP-IR, were hybridized with pEPI-SFFV (6.5 kb) construct plasmid used as a hybridization probe that detects episomal and/or integrated transgenes, labelled with Fluorescein-12-2′-deoxy-uridine-5′-triphosphate (Roche). Plasmid hβ-S/MAR (13.3 kb) plasmid was used as a hybridisation probe labelled with Tetramethyl-Rhodamine-5-dUTP (Roche) that detects the endogenous HBB locus. The procedure is reported in detail previously^30^. Probe labelling was performed by Nick Translation Mix (Roche) following the manufacturer’s instructions. A total of 100 metaphase and interphase spreads were analysed.

### Colony PCR

Fluorescent (eGFP^+^) and non fluorescent (eGFP^−^) colonies, from transfected CD34^+^ cells with pEPI-EF1/HTLV, pEPI-SFFV and pEP-IR were individually picked under a light microscope, mixed into two different pools and washed twice in 1 × PBS. Each pool contains at least 90 colonies. Total DNA was isolated with AllPrep DNA/RNA/Protein Mini Kit (Qiagen) and subsequently treated with RNase I (Promega). PCR reaction were performed using 400–500 ng genomic DNA quantified with NanoProp (Quawell), 0.25 u Taq polymerase (Invitrogen), 0.5 pmol dNTPs (Fermentas) and 0.25 pmol of each specific primer (Invitrogen). PCR primers were: eGFP_Forward: 5′-CAG CCA CAA CGT CTA TAT CAT G-3′, eGFP_Reverse: 5′-CTT GTA CAG CTC GTC CAT GC-3′, β-globin_Forward: 5′-GAA GAG CCA AGG ACA GGT-3′ and β-globin_Reverse: AGT TCA TGT CAT AGG AAG GGG GA-3′. PCR conditions were: denaturation at 95 °C for 7 min followed by 38 cycles (95 °C for 30 sec, 58 °C for 40 sec, 72 °C for 90 sec) and a final extension 72 °C for 7 min.

### Plasmid Copy Number estimation

Total DNA (50–100 ng) was isolated as described above from eGFP^+^ colonies derived from CD34^+^ cells transfected with pEPI-EF1/HTLV, pEPI-SFFV and pEP-IR. For qPCR, DNA was amplified using QuantyiFast SYBER Green PCR (Qiagen) and the LightCycler2.0 (Roche) as follows: 10 sec at 95 **°**C, 30 sec at 60 **°**C, 40 cycles. The eGFP DNA primers used were: eGFP_F 5′-GAC CAC TAC CAG CAG AAC AC-3′ and eGFP_R 5′-GAA CTC CAG CAG GAC CAT G-3′ (Invitrogen). Copy number estimation was carried out using the Absolute Quantification analysis (Light Cycler Software 4.05, Roche), following instructions provided by creating Standard Curves with Genomic DNA or Plasmid DNA Templates for Use in Quantitative PCR. Applied Biosystems. 2003. Revision A. Web PDF. 1 July 2011. Available at: http://www3.appliedbiosystems.com/cms/groups/mcb_marketing/documents/generaldocuments/cms_042486.pdf. In short, the mass of one copy of each plasmid was calculated using the form m = n.1.096e-21g/bp (where m = mass, n = genome plasmid size in bp, e-21 = x10^−21^). Then, from a plasmid DNA solution of 200 ng/μl, a series of dilutions were prepared, for 10, 10^2^, 10^3^, 10^4^, 10, 10^6^ copies per reaction. These dilutions were used in RT-qPCR with eGFP-specific primers, to produce a curve for every copy-number dilution of plasmid. Plasmid copy number in the DNA prepared from transfected cells was measured against these curves and the plasmid copy numbers **per cell** was calculated from these measures, on the basis that the mass of the diploid human genome is 6.6 pg.

### RNA Quantification

Total RNA (800 ng) from K562 cells and eGFP^+^ colonies derived from CD34^+^ cells transfected with pEPI-EF1/HTLV, pEPI-SFFV and pEP-IR, was treated with DNase I (Promega), and reverse transcribed to cDNA with Prime-Script TM, first-strand cDNA Synthesis Kit with Oligo dT primers (Takara, Japan). For qPCR, 1/50 of the cDNA were amplified using QuantiFast SYBR Green PCR (Qiagen) and the LightCycler2.0 (Roche) using primers eGFP_F and eGFP_R (see above) as follows: 10 sec at 95 °C, 30 sec at 58 °C, 40 cycles. For data normalization, the GAPDH gene was used as internal control. The GAPDH primers were: GAPDH_F 5′-CCA TGT TCG TCA TGG GTG TGA-3′and GAPDH_R 5′CAT GGA CTG TGG TCA TGA GT-3′ (Invitrogen).

### Western blotting

Total cell lysates were prepared by lysing cell pellets directly in SDS/PAGE loading buffer (50 mM Tris-HCl pH 6.8, 2% SDS, 10% glycerol, 50 mM dithiothreitol and 0.1% bromophenol blue) and boiling. All protein extracts were resolved in a 10% SDS-PAGE gel and electrotransferred to a polyvinylidene difluoride (PVDF) membrane (Millipore). eGFP protein was detected with anti-GFP rabbit antibody 1/500 (SantaCruz Biotechnology) and the endogenous tubulin with anti-tubulin mouse antibody 1/10000 (Sigma-Aldrich-Chemical Co.).

### Plasmid rescue

Extrachromosomal DNA was isolated from 90–120 colonies derived from FACS-sorted CD34^+^/eGFP^+^ cells transfected with pEP-IR vector, randomly collected, pooled and washed in 1xPBS as previously described, using a modified HIRT protocol^19^. The HIRT extract was used for transformation of *E. coli* ElectroMax DH5a-ETM cells (Invitrogen), by electroporation according to the manufacturer’s instructions and transformed *E. coli* colonies were selected using LB-agar plates containing 30 μg/ml kanamycin (Invitrogen). Plasmid DNA was extracted (QIAprep Spin Miniprep Kit, Qiagen) from randomly selected single resistant colonies and was subjected to restriction enzyme analysis with *AseI, XmnI* and *AvrII* enzymes. In total, 6 randomly picked colonies from three independent experiments (with 3, 2 and 5 colonies per dish respectively) were analyzed.

### Mouse thalassaemic bone marrow cell (BMC) transfection and flow cytometry assay

Mouse thalassaemic BMCs derived from 14-week-old Hbb^th-3/+^ donors and age-matched wild-type BMCs of C57BL/6 J donors (5 × 10^7^ for each cases) were transfected with vector pEP-IR (12 μg) by lipofection (Lipofectamine 2000, Invitrogen) according to the manufacturer’s instructions. Flow cytometric assay was applied 24 hours after transfection. Sca-1 Antibody (PE Rat Anti-Mouse Ly-6A/E, BD Biosciences) was used in combination with APC Mouse Lineage Antibody Cocktail (with isotype control, BD Biosciences) to identify haematopoietic progenitors in mouse marrow in flow cytometric assay. Dead cells were excluded from the assay after treatment with 7-amino actinomycin D (7-AAD) (BD Biosciences). Specifically, 5 × 10^5^ transfected and non-transfected cells were treated with 1 μl ScaPE antibody and 10 μl Lin APC antibody and stored at 4 **°**C in the dark for 15 min. Then 5 μl 7AAD were added and cells were stored again at 4 **°**C in dark for 5 min immediately before analysis.

### Statistical analysis

All comparisons among cell colonies were performed using non- parametric tests (Kruskal Wallis) and statistical significance was denoted with p values < 0.05.

## Additional Information

**How to cite this article**: Stavrou, E. F. *et al*. The β-globin Replicator greatly enhances the potential of S/MAR based episomal vectors for gene transfer into human haematopoietic progenitor cells. *Sci. Rep.* **7**, 40673; doi: 10.1038/srep40673 (2017).

**Publisher's note:** Springer Nature remains neutral with regard to jurisdictional claims in published maps and institutional affiliations.

## Supplementary Material

Supplementary Information

## Figures and Tables

**Table 1:** eGFP positive CFCs derived from FACS-sorted CD34^+^/eGFP^+^ cells, transfected with control vector pEPI-eGFP and the three experimental vectors: pEPI-EF1/HTLV, pEPI-SFFV and pEP-IR.

	BFU-E				CFU-GEMM				CFU-GM				Total			
	n	μ	μ	s.d (%)	n	μ	μ	s.d (%)	n	μ	μ	s.d (%)	n	μ	μ	s.d (%)
pEPI-eGFP	0/167	0/100.3	0	0	0/13	0/9.8	0	0	0/7	0/5.8	0	0	0/187	0/115.8	0	0
	0/85				0/2				0/3				0/90			
	0/33				0/6				0/4				0/43			
	0/116				0/18				0/9				0/143			
pEPI-EF1/HTLV	26/68	51.3/96.3	51.8	12.2	0/1	0/2	0	0	0/1	0/2	0	0	26/70	51.3/100.3	49.8	12
	79/120				0/1				0/2				79/123			
	48/84				0/3				0/1				48/88			
	52/113				0/3				0/4				52/120			
pEPI-SFFV	53/87	54.2/90.2	59.9	14.5	0/0	0/0	0	0	1/8	2/7.8	24.2	14.0	54/95	56.2/98	56.9	13.7
	36/68				0/0				1/6				37/71			
	79/120				0/0				4/9				83/135			
	38/94				0/0				3/7				40/98			
	65/82				0/0				3/9				68/91			
pEP-IR	190/190	149.8/149.8	100	0	0/0	0/0	0	0	7/7	4.2/4.2	100	0	197/197	154/154	100	0
	76/76				0/0				1/1				77/77			
	138/138				0/0				2/2				140/140			
	171/171				0/0				5/5				176/176			
	174/174				0/0				6/6				180/180			

n depicts the fraction of eGFP^+^ colonies over total number of colonies per dish from 4 to 5 transfection experiments, μ depicts the average fraction of these experiments.

## References

[b1] Romano G. Development of safer gene delivery systems to minimize the risk of insertional mutagenesis-related malignancies: a critical issue for the field of gene therapy. ISRN Oncol. 2012, 616310, doi: 10.5402/2012/616310 (2012). 23209944 PMC3512301

[b2] Piechaczek C., Fetzer C., Baiker A., Bode J. & Lipps H. J. A vector based on the SV40 origin of replication and chromosomal S/MARs replicates episomally in CHO cells. Nucleic acids research 27, 426–428 (1999). 9862961 10.1093/nar/27.2.426 PMC148196

[b3] Marie C. . pFARs, plasmids free of antibiotic resistance markers, display high-level transgene expression in muscle, skin and tumour cells. The journal of gene medicine 12, 323–332, doi: 10.1002/jgm.1441 (2010). 20209487

[b4] Gracey Maniar L. E. . Minicircle DNA vectors achieve sustained expression reflected by active chromatin and transcriptional level. Molecular therapy: the journal of the American Society of Gene Therapy 21, 131–138, doi: 10.1038/mt.2012.244 (2013). 23183534 PMC3538319

[b5] Verghese S. C., Goloviznina N. A., Skinner A. M., Lipps H. J. & Kurre P. S/MAR sequence confers long-term mitotic stability on non-integrating lentiviral vector episomes without selection. Nucleic Acids Res 42, e53, doi: 10.1093/nar/gku082 (2014). 24474068 PMC3985655

[b6] Jin C. . Safe engineering of CAR T cells for adoptive cell therapy of cancer using long-term episomal gene transfer. EMBO Mol Med 8, 702–711, doi: 10.15252/emmm.201505869 (2016). 27189167 PMC4931286

[b7] Kymalainen H. . Long-term episomal transgene expression from mitotically stable integration-deficient lentiviral vectors. Hum Gene Ther 25, 428–442, doi: 10.1089/hum.2013.172 (2014). 24483952 PMC4027990

[b8] Stehle I. M. . Establishment and mitotic stability of an extra-chromosomal mammalian replicon. BMC Cell Biology 8, 33 (2007). 17683605 10.1186/1471-2121-8-33 PMC1959191

[b9] Hagedorn C., Baiker A., Postberg J., Ehrhardt A. & Lipps H. J. A colony-forming assay for determining the establishment efficiency of S/MAR-containing nonviral episomal expression vectors. Cold Spring Harbor protocols 706–708, doi: 10.1101/pdb.prot069500 (2012). 22661431

[b10] Papapetrou E. P., Zoumbos N. C. & Athanassiadou A. Genetic modification of hematopoietic stem cells with nonviral systems: past progress and future prospects. Gene Ther 12 Suppl 1, S118–130, doi: 10.1038/sj.gt.3302626 (2005). 16231044

[b11] Chow C. M. . LCR-mediated, longterm tissuespespecific gene expression within replicating episomal plasmid and cosmid vectors. Gene Therapy 9(5), 327–36 (2002). 11938452 10.1038/sj.gt.3301654

[b12] Bode J. . Scaffold/matrix-attached regions: structural properties creating transcriptionally active loci. International review of cytology 162A, 389–454 (1995). 8575884 10.1016/s0074-7696(08)61235-8

[b13] Jenke B. H. . An episomally replicating vector binds to the nuclear matrix protein SAF-A *in vivo*. EMBO reports 3, 349–354, doi: 10.1093/embo-reports/kvf070 (2002). 11897664 PMC1084058

[b14] Stehle I. M., Scinteie M. F., Baiker A., Jenke A. C. & Lipps H. J. Exploiting a minimal system to study the epigenetic control of DNA replication: the interplay between transcription and replication. Chromosome research: an international journal on the molecular, supramolecular and evolutionary aspects of chromosome biology 11, 413–421 (2003). 10.1023/a:1024962308071 12971718

[b15] Baiker A. . Mitotic stability of an episomal vector containing a human scaffold/matrix-attached region is provided by association with nuclear matrix. Nature cell biology 2, 182–184, doi: 10.1038/35004061 (2000). 10707091

[b16] Glover D. J., Lipps H. J. & Jans D. A. Towards safe, non-viral therapeutic gene expression in humans. Nature reviews. Genetics 6, 299–310, doi: 10.1038/nrg1577 (2005). 15761468

[b17] Schaarschmidt D., Baltin J., Stehle I. M., Lipps H. J. & Knippers R. An episomal mammalian replicon: sequence-independent binding of the origin recognition complex. The EMBO journal 23, 191–201, doi: 10.1038/sj.emboj.7600029 (2004). 14685267 PMC1271667

[b18] Jenke A. C. . Nuclear scaffold/matrix attached region modules linked to a transcription unit are sufficient for replication and maintenance of a mammalian episome. Proceedings of the National Academy of Sciences of the United States of America 101, 11322–11327, doi: 10.1073/pnas.0401355101 (2004). 15272077 PMC509201

[b19] Papapetrou E. P., Ziros P. G., Micheva I. D., Zoumbos N. C. & Athanassiadou A. Gene transfer into human hematopoietic progenitor cells with an episomal vector carrying an S/MAR element. Gene therapy 13, 40–51, doi: 10.1038/sj.gt.3302593 (2006). 16094410

[b20] Argyros O. . Persistent episomal transgene expression in liver following delivery of a scaffold/matrix attachment region containing non-viral vector. Gene therapy 15, 1593–1605, doi: 10.1038/gt.2008.113 (2008). 18633447

[b21] Manzini S. . Genetically modified pigs produced with a nonviral episomal vector. Proceedings of the National Academy of Sciences of the United States of America 103, 17672–17677, doi: 10.1073/pnas.0604938103 (2006). 17101993 PMC1635978

[b22] Broll S., Oumard A., Hahn K., Schambach A. & Bode J. Minicircle performance depending on S/MAR-nuclear matrix interactions. Journal of molecular biology 395, 950–965, doi: 10.1016/j.jmb.2009.11.066 (2010). 20004666

[b23] Haase R. . pEPito: a significantly improved non-viral episomal expression vector for mammalian cells. BMC biotechnology 10, 20, doi: 10.1186/1472-6750-10-20 (2010). 20230618 PMC2847955

[b24] Ehrhardt A. . Episomal vectors for gene therapy. Current gene therapy 8, 147–161 (2008). 18537590 10.2174/156652308784746440

[b25] Voigtlander R. . A Novel Adenoviral Hybrid-vector System Carrying a Plasmid Replicon for Safe and Efficient Cell and Gene Therapeutic Applications. Molecular therapy. Nucleic acids 2, e83, doi: 10.1038/mtna.2013.11 (2013). 23549553 PMC3650243

[b26] Schubeler D., Mielke C., Maass K. & Bode J. Scaffold/matrix-attached regions act upon transcription in a context-dependent manner. Biochemistry 35, 11160–11169, doi: 10.1021/bi960930o (1996). 8780520

[b27] Giannakopoulos A. . The functional role of S/MARs in episomal vectors as defined by the stress-induced destabilization profile of the vector sequences. Journal of molecular biology 387, 1239–1249, 10 (2009). 19248788 10.1016/j.jmb.2009.02.043

[b28] Aladjem M. I. The mammalian beta globin origin of DNA replication. Frontiers in bioscience: a journal and virtual library 9, 2540–2547 (2004). 15358579 10.2741/1415

[b29] Wang L., Lin C. M., Lopreiato J. O. & Aladjem M. I. Cooperative sequence modules determine replication initiation sites at the human beta-globin locus. Human molecular genetics 15, 2613–2622, doi: 10.1093/hmg/ddl187 (2006). 16877501

[b30] Sgourou A., Routledge S., Spathas D., Athanassiadou A. & Antoniou M. N. Physiological levels of HBB transgene expression from S/MAR element-based replicating episomal vectors. Journal of biotechnology 143, 85–94, doi: 10.1016/j.jbiotec.2009.06.018 (2009). 19559736

[b31] Sirven A. . Enhanced transgene expression in cord blood CD34(+)-derived hematopoietic cells, including developing T cells and NOD/SCID mouse repopulating cells, following transduction with modified trip lentiviral vectors. Molecular therapy: the journal of the American Society of Gene Therapy 3, 438–448, doi: 10.1006/mthe.2001.0282 (2001). 11319904

[b32] Demaison C. . High-level transduction and gene expression in hematopoietic repopulating cells using a human immunodeficiency [correction of imunodeficiency] virus type 1-based lentiviral vector containing an internal spleen focus forming virus promoter. Human gene therapy 13, 803–813, doi: 10.1089/10430340252898984 (2002). 11975847

[b33] Bode J. . Correlations between scaffold/matrix attachment region (S/MAR) binding activity and DNA duplex destabilization energy. Journal of molecular biology 358, 597–613, doi: 10.1016/j.jmb.2005.11.073 (2006). 16516920

[b34] Hagedorn C., Baiker A., Postberg J., Ehrhardt A. & Lipps H. J. A colony-forming assay for determining the establishment efficiency of S/MAR-containing nonviral episomal expression vectors. Cold Spring Harb Protoc 2012, 706–708, doi: 10.1101/pdb.prot069500 (2012). 22661431

[b35] Yang B. . O. A mouse model for β0-thalassemia. Proc. Natl. Acad. Sci. USA 92, 11608-1 1612 (1995).

[b36] Okada N. & Shimizu N. Dissection of the beta-globin replication-initiation region reveals specific requirements for replicator elements during gene amplification. PLoS One 8, e77350, doi: 10.1371/journal.pone.0077350 (2013). doi: 10.1016/j.jmb.2009.02.043 (2009). PMC3790722 24124615

[b37] Merkenschlager M. & Odom D. T. CTCF and cohesin: linking gene regulatory elements with their targets. Cell 152, 1285–1297, doi: 10.1016/j.cell.2013.02.029 (2013). 23498937

[b38] Zielke K. . The insulator protein CTCF binding sites in the orf73/LANA promoter region of herpesvirus saimiri are involved in conferring episomal stability in latently infected human T cells. Journal of virology 86, 1862–1873, doi: 10.1128/JVI.06295-11 (2012). 22130528 PMC3264369

[b39] Hagedorn C., Antoniou M. N. & Lipps H. J. Genomic cis-acting Sequences Improve Expression and Establishment of a Nonviral Vector. Molecular therapy. Nucleic acids 2, e118, doi: 10.1038/mtna.2013.47 (2013). 24002728 PMC3759742

[b40] Saunders F., Sweeney B., Antoniou M. N., Stephens P. & Cain K. Chromatin Function Modifying Elements in an Industrial Antibody Production Platform-Comparison of UCOE, MAR, STAR and cHS4 Elements. PLoS One 10, 4 (2015). 10.1371/journal.pone.0120096 PMC4388700 25849659

[b41] Bi C. & Benham C. J. WebSIDD: server for predicting stress-induced duplex destabilized (SIDD) sites in superhelical DNA. Bioinformatics 20, 1477–1479, doi: 10.1093/bioinformatics/bth304 (2004). 15130924

