# Supplementary material for: The β-globin Replicator greatly enhances the potential of S/MAR based episomal vectors for gene transfer into human haematopoietic progenitor cells
Source: Sci Rep. 2017 Jan 20;7:40673. doi: 10.1038/srep40673 (PMC5247744; doi:10.1038/srep40673)
Supplement: Supplementary Information [file srep40673-s1.pdf]

Supplementary Information

Title:

The β-globin Replicator greatly enhances the potential of S/MAR based episomal vectors for gene transfer into human haematopoietic progenitor cells

Authors list:

Eleana F. Stavrou, Vassileios M. Lazaris, Aristeidis Giannakopoulos, Eirini Papapetrou, Alexandros Spyridonidis, Nikolas C. Zoumbos, Antonis Gkountis, Aglaia Athanassiadou

Suppl Figure S0. IR region

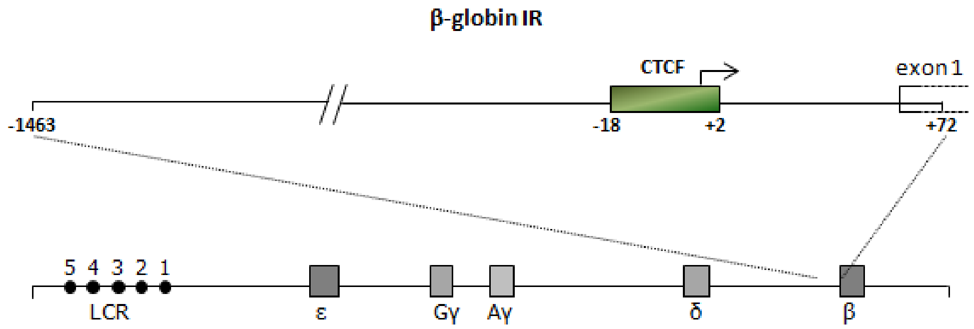

The part of the β-globin Replicator (IR) used in this study, containing the 1.3 kb that represents the consensus IR region, is shown enlarged above the β-globin gene cluster depicting all five β-globin-like genes and the locus control region (LCR), spanning in total 80 kb on chromosome 11. Arrow denotes beginning of transcription of the β-globin gene. CTCF: position of the consensus binding site for protein CTCF. Exon 1: the beginning of exon 1 of the β-globin gene.

Suppl Figure S1. eGFP expression in transfected K562 cells

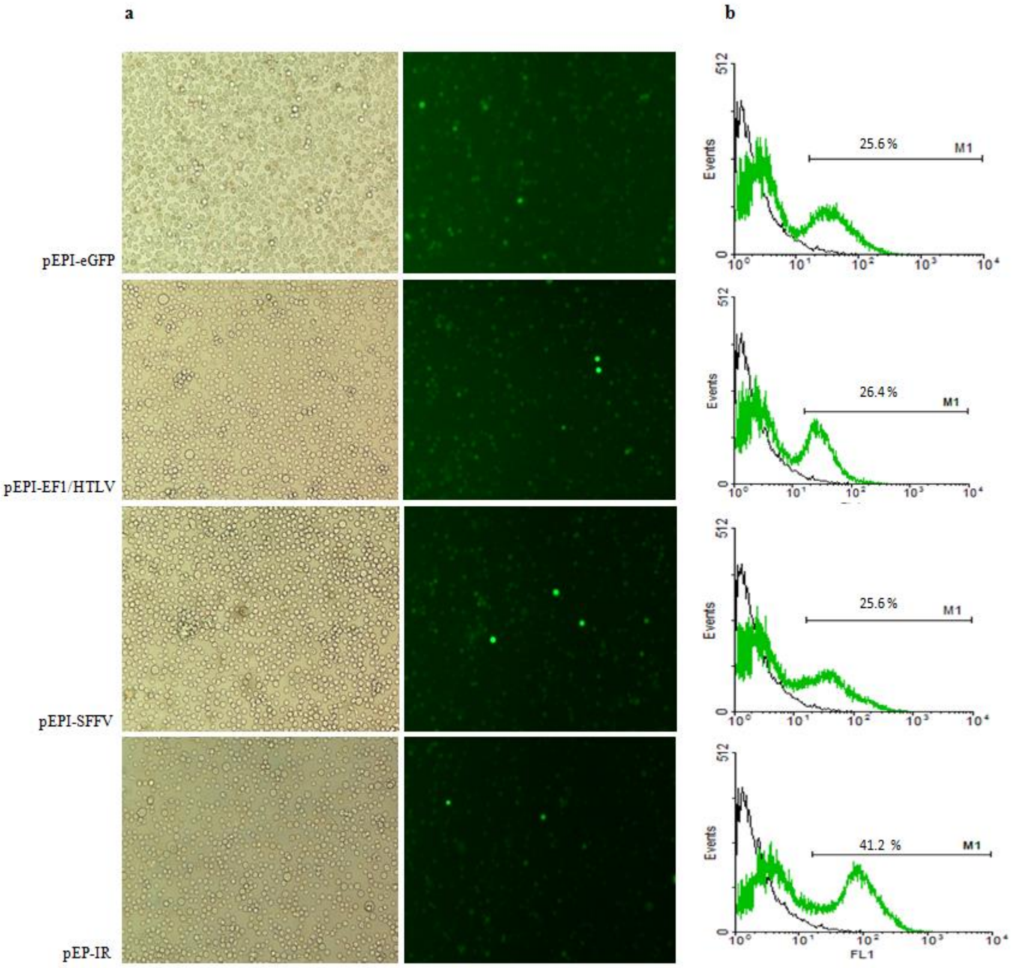

Documentation of eGFP expression in transfected K562 cells carrying the control pEPI-eGFP vector and each of the three experimental vectors, 48 hours after transfection. Results of one representative experiment are shown for (a) phase contrast (left column) and fluorescent microscopy (right column) and (b) flow cytometry, giving the estimated percentage of fluorescent, transfected cells for each vector used. y-axis records counts of cells and x-axis records FL1-eGFP fluorescence.

Supplementary Figure S2. Resistance test in transfected K562 cells

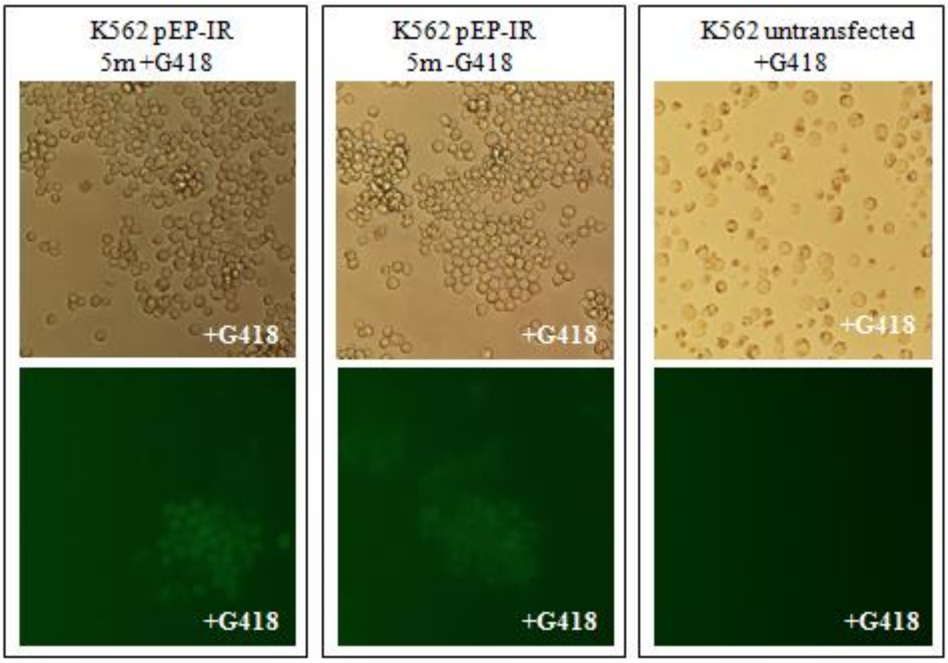

K562 cells after the addition of 400 ng/ml antibiotic G418, depicting K562 cells carrying the experimental pEP-IR vector 5 months post-transfection in continuous culture with 400 ng/ml antibiotic (5m+G418) (left) and the same cells without antibiotic (5m-G418) (middle), and untransfected K562 (right) with antibiotic. Phase contrast photographs (upper line of photos) and fluorescent microscope photographs (lower line of photos).

Supplementary Figure S3. Mean fluorescence intensity in transfected K562 cells

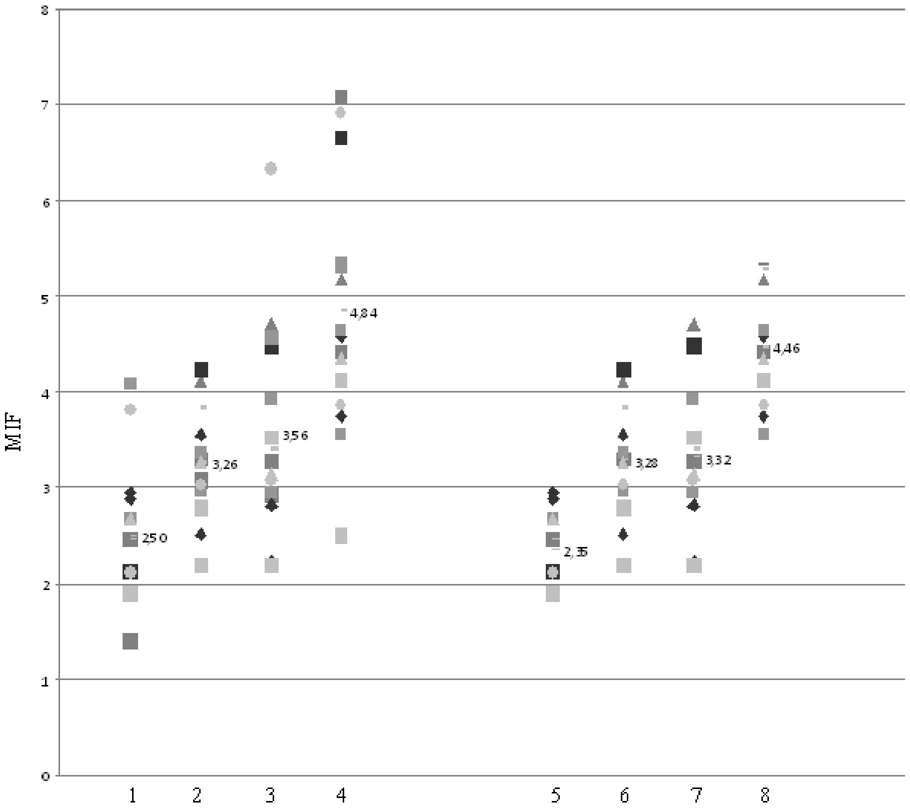

Mean fluorescence intensity measurements, 3 months (numbers 1-4) and 5 months (numbers 5-8) post-transfection with vectors pEPI-eGFP (numbers 1 and 5), pEPI-EF1/HTLV (numbers 2 and 6), pEPI-SFFV (numbers 3 and 7) and pEP-IR (numbers 4 and 8). The data shown derive from three independent experiments and cultures with and without antibiotic selection. The average value is shown in each case.

Supplementary Figure S4. BMCs transfected with pEP-IR

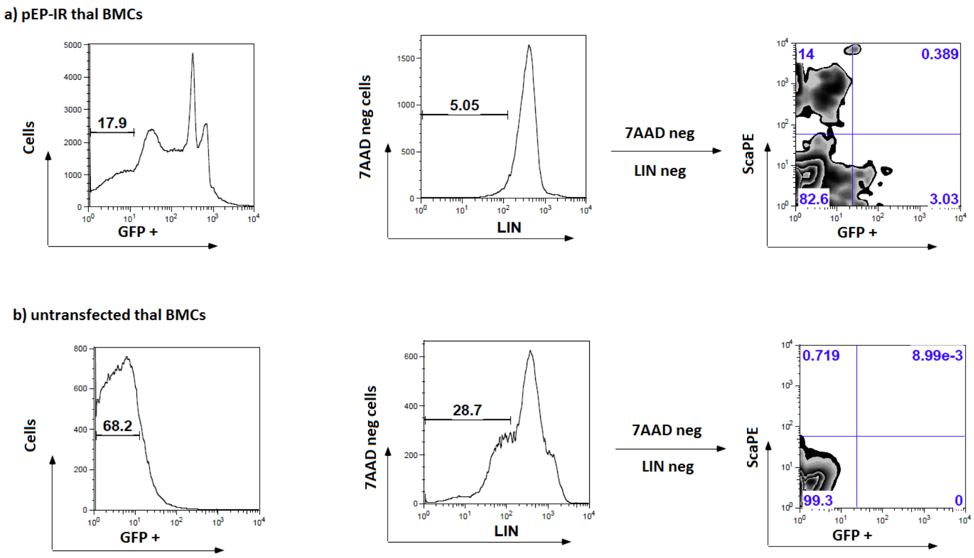

pEPI-IR is capable for transfecting β-thalassaemia mouse progenitor cells as shown at this indicative experiment. Upper line of panels refer to mouse β-thalassaemia Bone Marrow Cells (BMCs) transfected with pEP-IR and down line of panels refer to untransfected, thalassaemic mouse BMCs. Flow cytometric analysis panels indicate: (a) 7-AAD$^-$ live cells, (b) selected cell populations for analysis based on the cell size, (c) Lin$^-$ cells from eGFP$^+$ selected cell population, (d) ScaPE$^+$ cells from eGFP$^+$ selected cell population, (e) 7-AAD$^-$ cells from Lin$^-$ population and (f) eGFP$^+$/Lin$^-$/Sca1$^+$ cells. In (c), (d) and (f) panels all cells analyzed are 7AAD negative.

Suppl Figure S5. eGFP and tubulin detection in Western blot analysis (Fig. 4b)

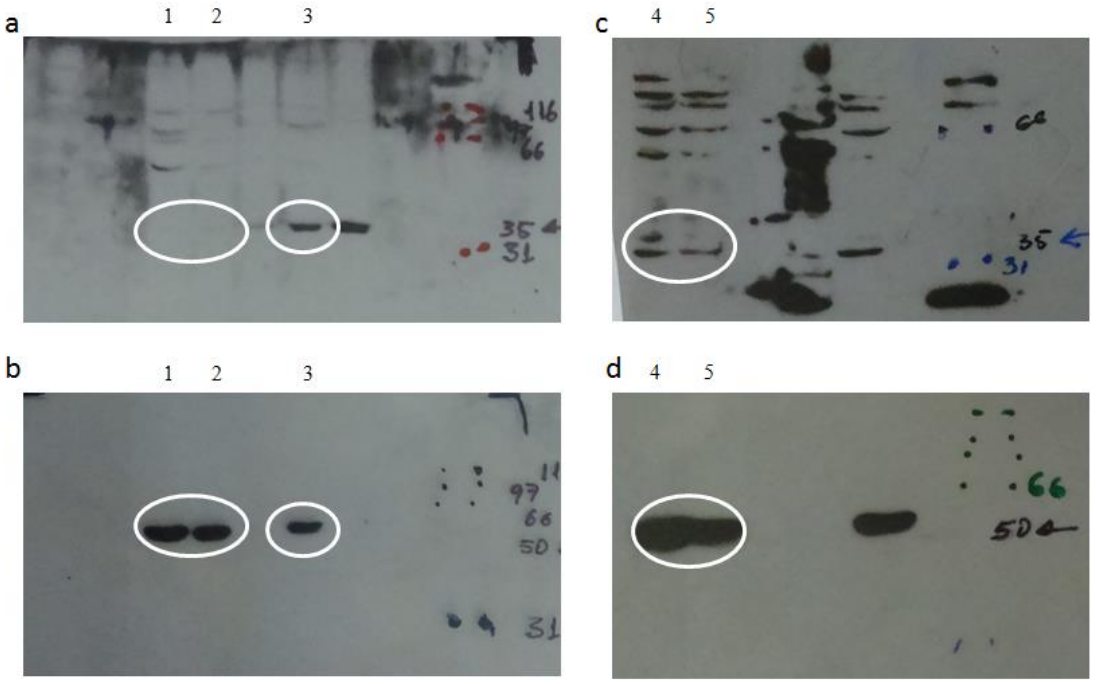

(a) eGFP and (b) tubulin detection for samples (cells from CFC culture colonies) 1. untransfected cells; 2. cells transfected with pEPI-eGFP; 3. cells transfected with pEP-IR; (c) eGFP and (d) tubulin detection for samples 4. cells transfected with pEPI-SFFV; 5. cells transfected with pEPI-EF1/HTLV.
